# The Gut Bacteria of *Gampsocleis gratiosa* (Orthoptera: Tettigoniidae) by Culturomics

**DOI:** 10.3390/insects16020123

**Published:** 2025-01-27

**Authors:** Hongmei Li, Huimin Huang, Ying Jia, Yuwei Tong, Zhijun Zhou

**Affiliations:** 1 Key Laboratory of Zoological Systematics and Application of Hebei Province, College of Life Sciences, Hebei University, Baoding 071002, China; lhmsdjn@163.com (H.L.); 17862061092@163.com (H.H.); jia13102022@163.com (Y.J.); t17636268216@163.com (Y.T.); 2 Institute of Life Science and Green Development, Hebei University, Baoding 071002, China

**Keywords:** *Gampsocleis gratiosa*, culturomics, gut bacteria

## Abstract

*Gampsocleis gratiosa* Brunner von Wattenwyl, 1862, is a type of omnivorous chirping insect with a long history of artificial breeding. It has high economic value and is also an excellent orthopteran model organism. In this study, 12 types of culture media combined with 16S rRNA sequencing were employed to isolate 838 bacterial strains from the gut of *G. gratiosa*. After sequence comparison, a total of 98 species of bacteria were identified, belonging to 3 phyla, 5 classes, 11 orders, 20 families, and 45 genera. Firmicutes and Proteobacteria accounted for the majority (92.86%). At the order level, Enterobacteriaceae, Bacillales, and Lactobacillales predominated (79.59%). At the genus level, *Klebsiella* (11.22%) and *Enterococcus* (7.14%) predominated. This study also enumerated the strain morphological, physiological and biochemical properties of 98 species of bacteria, including colony morphology, Gram staining, bacterial motility test, temperature gradient growth, pH gradient growth, citrate utilization test, temperature oxidase test, contact enzyme test, methyl red test, V-P test, indole test, gelatin liquefaction test, nitrate reduction test, hydrogen sulfide test, starch hydrolysis test, cellulose decomposition test, esterase (corn oil) test and antibiotic susceptibility testing. Additionally, 16 antibiotics were utilized to test the bacterial susceptibility of the strains. This study explored the types and community structure of some culturable microorganisms in the intestinal tract of *G. gratiosa* and recorded their physiological characteristics. These data reflect the physiological functions of the intestinal microorganisms of *G. gratiosa* and provide support for subsequent research on the interaction mechanism between microorganisms and their hosts.

## Simple Summary

Many microorganisms exist in the gut of insects and are collectively referred to as the gut microbiota. *Gampsocleis gratiosa* (Orthoptera, Tettigoniidae) is an omnivorous chirping insect with a long history of artificial breeding in China. In the present study, the bacterial communities of laboratory-reared *G. gratiosa* feces were isolated and identified using 12 different media and we investigated the physiological and biochemical characteristics and the antibiotic susceptibility of the isolates to 16 common antibiotics, laying a theoretical foundation for the further exploration of the physiological function of gut bacteria of *G. gratiosa* and the relationship between gut bacteria and host.

## 1. Introduction

Insects have developed special digestive tract structures during the long course of evolution [1]. Many microorganisms, such as bacteria, fungi, yeasts, protists, archaea and viruses, exist in the gut of insects and are collectively referred to as the gut microbiome. Numerous studies have indicated that although the diversity of gut microbiota in most insects is lower than in vertebrates, the quantities are relatively large and specific [2]. With the advancement of science and technology, people are gradually recognizing the significance of gut microbiota. Research on the gut microbiome is imperative as it plays a crucial role in the host’s nutrition, growth, and adaption. The intestinal tract of insects offers a distinctive environment for microorganisms, which form a stable and relatively simplified community structure through long-term co-evolution with the host [3]. Additionally, the insect gut microbiome can also provide biological resources for various fields like medicine, agriculture and ecology, and has broad applications.

*Gampsocleis gratiosa* Brunner von Wattenwyl, 1862, belongs to the order Orthoptera, family Tettigoniidae. It is a common omnivorous song insect. It is an incomplete metamorphosis insect and undergoes three stages in its life. Due to its crisp and loud chirping, it is loved by Chinese chirping insect players and has high economic value.

Current research on *G. gratiosa* mainly focuses on molecular phylogeny [4], transcriptome [5], mitochondrial genome [6], gene cloning, and ultrastructure [7]. Relatively, there are few studies on the gut microbiome. Illumina 16S rDNA V3-V4 region sequencing technology has been employed to detect fecal microorganisms in *G. gratiosa* at different developmental stages and genders, and previously counted 2982 OTUs, which were assigned into 25 phyla, 42 classes, 60 orders, 116 families, 241 genera, and some unclassified groups. A total of 21 core OTUs were classified into three phyla, four classes, five orders, eight families, and thirteen genera [8]. There were differences in the composition of gut bacteria at different stages of development when the feeding conditions were the same, which means the proportion of each gut bacteria was related to the course of host development and sex, and physiological changes in the host affect the growth of microorganisms.

However, there are limitations in studying the structure of intestinal microbial communities using only 16S gene sequencing. Although gene sequencing can reveal the diversity of microbial communities, it is difficult to accurately classify them to the species level [9], and it is impossible to culture and observe unknown microorganisms [10], which also hinders the exploration of the application of microbial resources. Culture-dependent methods are indispensable for understanding the species and diversity, ecological role, physiological function, metabolic mode and the symbiotic relationship with the host of insect intestinal bacteria, and are a necessary way to discover new species, new genes and new functions of bacteria [11].

Culturomics is a method developed in recent years for cultivating and identifying unknown bacteria using a combination of multiple culture conditions and rapid identification. Unlike pyrosequencing, culturomics emphasize systematically applying a large sample of culture conditions, isolating as many individual microorganisms as possible through different culture conditions [12,13]. Culturomics techniques have also been applied to isolate and culture insect gut bacterial, such as *Bactrocera tau* [14], *Diuraphis noxia* [15], and *Cotesia vestalis* [16].

In current research, there have been an increasing number of studies on the composition, diversity and function of intestinal bacteria of Orthoptera insects, among which the Acridoidea and Cricket superfamilies have received the most in-depth study. Smith et al. conducted a metagenomic high-resolution analysis of the gut bacteria community structure of *Anabrus simplex* and found that its bacteria were mainly composed of Lactobacilliaceae, Streptococcaceae and Enterobacteriaceae. They also isolated 13 bacterial strains using tryptone soybean agar medium, brain heart infusion agar medium, nutrient agar medium and MRS agar medium, which were identified as Lactobacilliaceae and Enterobacteriaceae [17].

In general, there is a significant difference between the bacterial flora obtained by pure culture methods and non-culture methods. Using the Illumina NovaSeq platform to study the diversity of intestinal bacteria in 12 species of Ensifera, it was found that the composition of gut bacteria in hosts with different feeding habits differed significantly [18]. The dominant bacteria of herbivorous and carnivorous insects is Proteobacteria, while that of omnivorous insects is Firmicutes. The study further showed that feeding habits and taxonomic status jointly affect the composition of intestinal bacterial flora [18]. When the taxonomic status is lower than the suborder level, the influence of feeding habits dominates. The intestinal bacterial diversity of omnivorous insects and carnivorous insects is higher than that of herbivorous insects, and the intestinal bacterial flora is not highly correlated with host taxonomic status at the phylum and genus levels. Zheng et al. analyzed the intestinal flora of 12 species of Ensifera through shotgun metagenomic sequencing, further elucidating the composition and function of the intestinal flora and its relationship with the feeding habits of the host. They showed that the diversity of the microbial community of herbivorous insects is higher than that of omnivorous and carnivorous insects, which is different from previous results that only compared the diversity of intestinal bacteria [19].

Current research on the intestinal microorganisms of Katydids is relatively scarce, and there are also few studies on the specific culture and physiological and biochemical characteristics of gut bacteria. This study combined the traditional bacterial isolation and 16S rRNA gene sequencing to culture and identify the gut bacteria of *G. gratiosa*, and further explored the morphological, physiological and biochemical characteristics of culturable bacteria.

## 2. Materials and Methods

### 2.1. Samples

Fifty adult *G. gratiosa* were bought from the Songbug Market in Baoding City, Hebei Province, China. Samples were placed in a laboratory artificial climate box (relative humidity 50%, temperature (30 ± 2) °C, photoperiod L:D = 12:12) and starved for 24 h to avoid interference from transient bacteria. We soaked samples in 75% alcohol for 1 min, and then washed them with deionized water to avoid the interference of bacteria on the surface of the insects. Samples were dissected with dissecting scissors; the gut was then isolated from other tissues and placed in a sterile 2 mL centrifuge tube. We added sterile water to 1000 µL and homogenized using a high-throughput tissue grinder. To avoid the contamination of microorganisms from the air in the laboratory, the above operations were carried out in a biosafety cabinet.

### 2.2. Culturomics

Culturomics is one high-throughput approach that multiplies culture conditions to detect greater bacterial diversity and obtain pure bacterial cultures [20]. This culturomics study encompassed 12 kinds of mediums (Table 1), which were obtained from the Leibniz Institute DSMZ-German Collection of Microorganisms and Cell Cultures by KOMODO (Known Media Database) media recommendation system [21]. Each culture medium comprised a solid type (for culturing colonies) and a liquid type (for enriching strains).

#### 2.2.1. Gradient Culture of Bacterial Suspension

We serially diluted the gut homogenate with sterile water to 10^−8^, and selected four gradients of bacterial suspensions with concentrations of 10^−2^, 10^−4^, 10^−6^,10^−8^ for coating culture. A total of 200 µL of each suspension was directly inoculated on various media and cultured at 30 °C for 48 h. For the medium for which colony growth was not evident, we extended the culture time appropriately. Each concentration of the gut homogenate was treated three times. We took photos to record the growth results of colonies.

#### 2.2.2. Isolation and Purification of Single Colonies

Colonies were picked based on the growth in the bacterial suspension culture plate. For colonies that were not clearly separated, the culture dish was divided into 16 or 9 grids for selective picking. After streaking the cultures for 24 h (extending the culture time if the growth of a single colony was not obvious), the morphological characteristics of the colonies on the streak plates were observed, and plate streaking was continued according to the culture conditions until an obvious single colony was isolated. The single colony was picked, cultured on a new plate and purified to obtain a single clone strain. A single bacterial colony was picked into a test tube containing liquid culture medium in a biosafety cabinet, shaking at 30 °C for 24 to 72 h until the bacterial liquid became turbid (1.0 ≤ OD600 ≤ 2.0). We took 1 mL of bacterial suspension into a sterile 2 mL centrifuge tube; an equal volume of sterile 40% glycerol solution was added, mixed thoroughly, sealed with parafilm, and refrigerated at −80 °C. The remaining bacterial suspension was used for DNA extraction and identification.

#### 2.2.3. Species Identification

Monoclonal genomic DNA was extracted with a TIANGEN Bacterial DNA kit (TIANGEN Biotech Beijing Co., Ltd., Beijing, China) according to the manufacturer’s instructions. The concentration and purity of DNA were determined by a NanoDrop 2000 UV-vis spectrophotometer (Thermo Scientific, Wilmington, NC, USA). The full length of bacterial 16S rRNA gene was amplified by polymerase chain reactions (the reaction procedure was as follows: 94 °C for 5 min, 34 cycles at 94 °C for 1 min, 50 °C for 1 min, 72 °C for 2 min and 72 °C for 7 min) with primers 27F (5′-AGAGTTTGATCCTGGCTCAG-3′) and 1492R (5′-TACGGCTACCTTGTTACGACTT-3′) [22], using a thermocycler PCR system. After PCR products were detected by 1% agarose gel electrophoresis, GENERAL BIOL (Anhui, China) was used to perform bidirectional sequencing. Sequencing results were processed through SeqMan; BLAST search and comparison were performed in the EzBioCloud database (http://www.ezbiocloud.net accessed on 1 December 2024 and GenBank database (https://blast.ncbi.nlm.nih.gov accessed on 1 December 2024) to obtain the species information with the highest similarity to the sequenced strains to initially determine the taxonomic status of the strains.

#### 2.2.4. Study on the Morphological Characteristics of the Gut Bacterial

The selected strains were re-streaked on the corresponding culture medium, colonies were observed, with their characteristics were recorded. The strain was cultured at 35 °C for 18 to 24 h. Colonies were picked and smears were prepared on clean glass slides. After natural drying, the slide was passed through the flame 2 to 3 times for fixation and cooled until it was no longer hot to the touch. Crystal Violet Ammonium Oxalate Solution was added to the fixed smear for staining 30 s. The staining solution was rinsed off slowly with water and the water was absorbed. Then, 95% ethanol was continuously added dropwise until the flowing liquid became colorless, and then rinsing with water was carried out immediately. Safranin O Stain Solution was added for 30 s, washed with water, and dried naturally. An oil lens was used to observe the smear. Gram-positive bacteria were purple (+) and Gram-negative bacteria were red (−). The motility of strains was tested using the semi-solid agar puncture method. The strains were picked out with sterile inoculation needles and inoculated into a test tube containing a semi-solid medium, then placed at 35 °C for 1 to 3 days, and the experimental results were visually observed. If the strain spreads and grows along the puncture line in a cloud-like manner, it indicates that the strain is motile (+); if the strain only grows along the puncture line with a clear edge, it indicates that it is non-motile (−).

#### 2.2.5. Physiological and Biochemical Properties

The physiological and biochemical characteristics of the isolated bacteria were tested according to the “Manual for Systematic Classification and Identification of Common Bacteria and Archaea” [23], which included citrate utilization test, temperature oxidase test, catalase test, methyl red test, V-P test, indole test, gelatin liquefaction test, nitrate reduction test, hydrogen sulfide test, starch hydrolysis test, cellulose decomposition test, esterase (corn oil) test and antibiotic susceptibility testing. Antibiotic sensitivity test was carried out by using the drug-sensitive paper inhibition zone method; 9 classes of 16 common antibiotics were used to test the drug sensitivity of the tested strains. The degree of resistance represented by the diameter of the inhibition zone of different antibiotics was different, and the diameter of inhibition zone reflected the sensitivity of strains to different antibiotics. The types of antibiotics and the criteria for antibiotic sensitivity are shown in Supplementary Table S1.

Growth at different pH for strains were also tested. We prepared liquid culture media with different pH values, including pH = 2, pH = 4, pH = 7, pH = 9 and pH = 11. The selected strains were mixed in 1 mL water and diluted to 10^−6^. Then, we pipetted 200 μL of the bacterial suspension into a centrifuge tube containing 1.5 mL of liquid culture medium. Each pH value was treated with three repetitions. A centrifuge tube containing 200 μL of water and 1.5 mL of liquid medium with pH 7 was used as a blank control group. The centrifuge tube was placed in a constant temperature metal bath, cultured at 220 rpm and 37 °C for 1~5 days. The turbidity of the bacterial solution was observed and recorded every 24 h. If the bacterial solution still showed no turbidity after 5 days, it was recorded that the target strain did not grow at this pH value.

## 3. Results

### 3.1. Bacterial Diversity of the Gut Bacteria Using Culturomics

**Figure 1:**
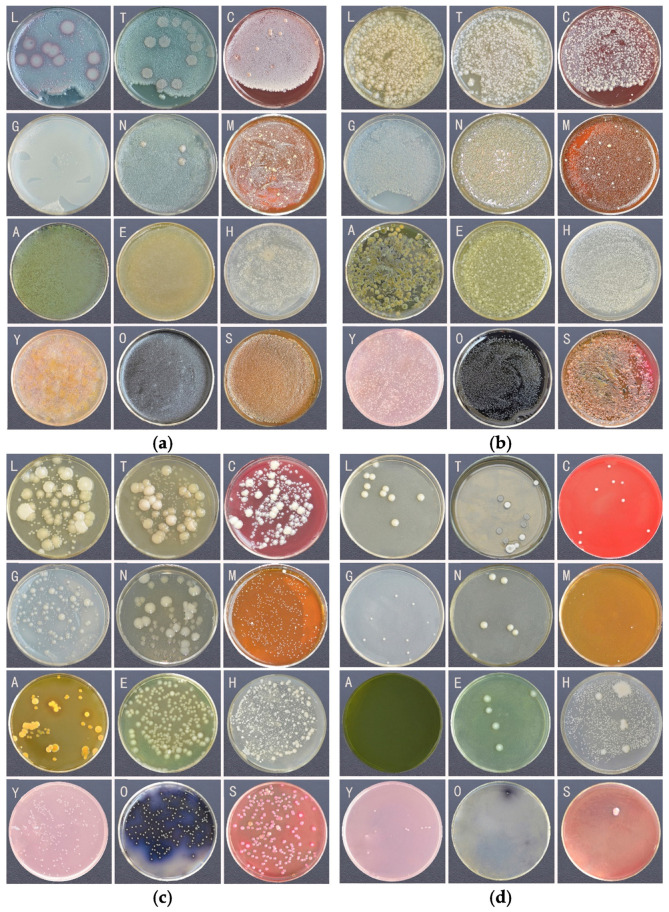
Culture results of gradient bacteria suspension in each culture medium. Different groups represent different concentrations of bacterial suspension. (**a**) 10^−2^; (**b**) 10^−4^; (**c**) 10^−6^; (**d**) 10^−8^. Different letters on the figure indicate different media. L: LB; T: TSA; C: BLA; G: GAO; N: NAM; M: MRS; A: AER; E:EE; H: CAR; Y: YCFA; O: ENT; S: SS.

The culture results of the gut bacteria are shown in Figure 1. The results indicated that the effect of different culture media on colony density was less than that of different bacterial suspension concentrations. The colonies in the culture medium inoculated with 10^−2^ and 10^−4^ concentration bacterial suspensions grew densely and single colonies could not be identified. Single colonies began to appear in the culture medium inoculated with 10^−6^ bacterial suspension, and there was little difference among different culture media. In the culture medium for culturing 10^−8^ bacterial suspension, there were colonies in GAO, YCAF and LB medium, while there was almost no colony growth in AER medium and SS agar.

**Figure 2:**
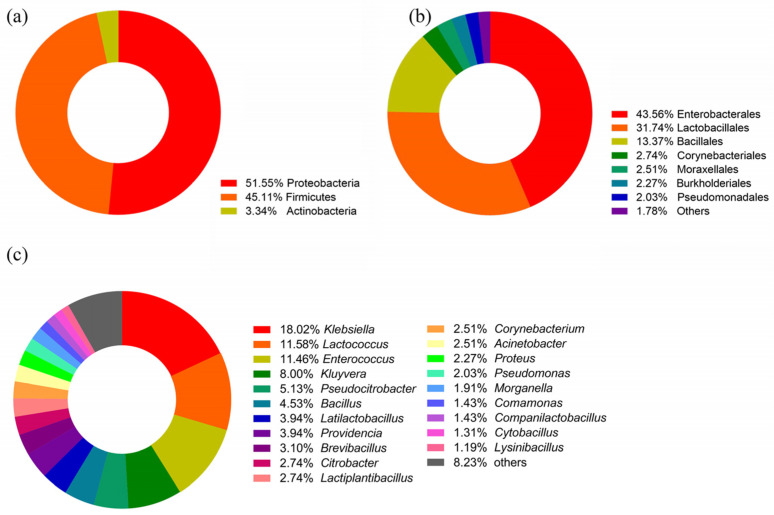
The classification results of 98 bacterial species. (**a**) Phylum level; (**b**) Order level; (**c**) Genus level.

After isolation and purification of the colonies on the culture medium, a total of 838 monoclonal strains were obtained. The species information of these strains was obtained through 16S rRNA sequence comparison. A total of 98 bacterial species were observed from 838 strains of intestinal bacteria (Supplementary Table S2). The classification results are shown in Figure 2. At the phylum level, the bacteria disseminated into Proteobacteria, Firmicutes, Actinobacteria (Figure 2a). At the order level, Enterobacterales, Lactobacillales, and Bacillales were dominant (Figure 2b). At the genus level, *Klebsiella* was relatively dominant, with a total of 151 strains isolated, followed by *Lactococcus*, *Enterococcus* and *Kluyveria*, with 97, 96 and 67 strains respectively. *Fictibacillus*, *Microbacterium*, *Shigella*, *Staphylococcus*, *Oceanobacillus*, *Enterobacter*, *Mammaliicoccus*, *Mycolicibacterium*, *Paenibacillus*, *Priestia*, *Serratia*, *Solibacillus*, *Agrococcus*, *Bordetella*, *Nocardiopsis*, *Pediococcus*, *Raoultella* and *Wohlfahrtiimonas* had less than three colonies (Figure 2c).

### 3.2. The Effect of Culture Conditions on Bacterial Culture

**Figure 3:**
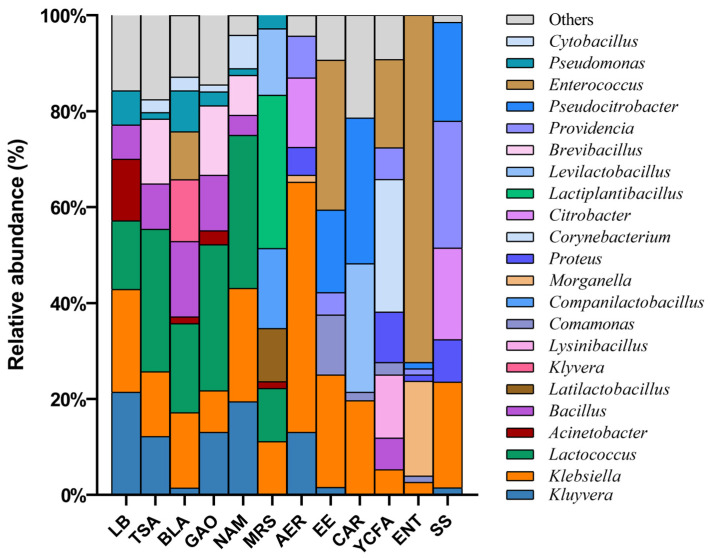
The distribution of genus-level bacteria in different media. The genus of which abundance is less than 1% in all samples were classified into “Other”.

The dominant bacteria in different culture media were also different; the distribution of genus-level bacteria in different media is shown in Figure 3. Comparing the bacterial strains isolated from different media, it was found that there were differences in the number of bacterial species isolated from different media. The bacteria isolated from COL, ACT and YCFA had rich diversity, including strains of three phyla. All of the bacteria isolated from AER and SS agar were Enterobacteriaceae, which was the narrowest range of isolates. The strains isolated from seven other culture media were Firmicutes and Proteobacteria, but no Actinobacteria.

The dominant bacteria in different culture media were also different. Klebsiella was distributed in 12 culture media, and Kluyvera was distributed in 8 culture media. In addition, 21 genera of bacteria were distributed in only one culture medium. There were two species of bacteria distributed in seven culture media, namely Klebsiella aerogenes and Kluyvera cryocrescens. A total of 57 species of intestinal bacteria were distributed in only one kind of culture medium.

**Figure 4:**
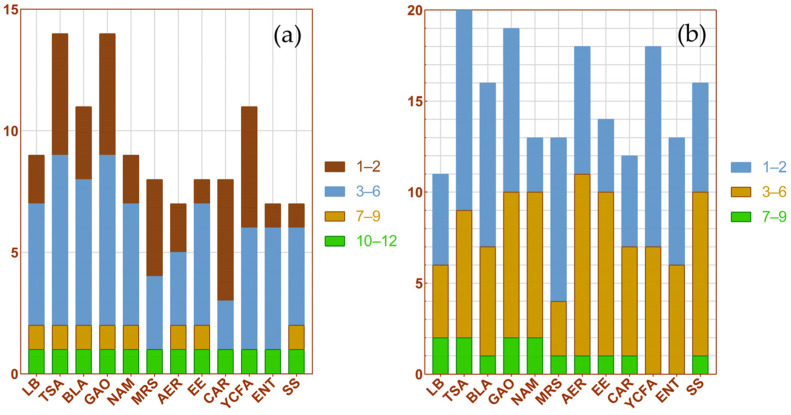
The number of strain types obtained from each culture medium. The y-axis represents the number of strain types, and the different colors indicate the extent of the distribution of strain in twelve media. (**a**) Genus level; (**b**) Species level.

The number of strain types obtained from each culture medium was counted at the genus and species levels. The results are shown in Figure 4a,b. Among the 12 kinds of culture media, TSA and GAO isolated the most types of bacteria, as about 15 genera and 20 species of bacteria could be isolated. TSA, MRS, CAR and YCFA isolated more bacteria that required special culture conditions, which were only distributed in one medium and accounted for more than half of the total number of culture media. In comparison, the number of bacteria cultured in LB medium and AER medium were smaller and the types were more common. In addition, *Klebsiella aerogenes* and *Kluyvera cryocrescens* did not appear in YCFA medium and ENT medium, while these two bacteria were almost distributed in other culture medium.

### 3.3. Morphological and Physiological and Biochemical Characteristics of Strains

#### 3.3.1. Physiological and Biochemical Characteristics

**Figure 5:**
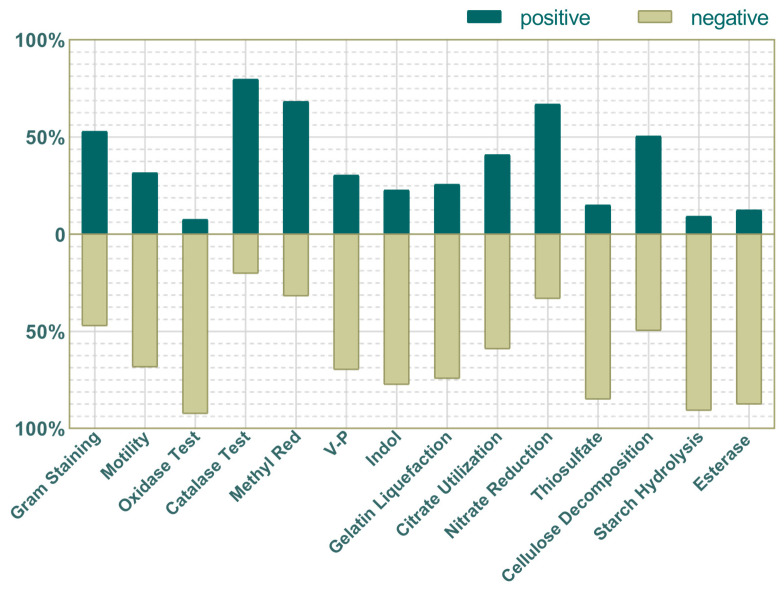
The morphological and physiological and biochemical characteristics of the identified bacteria. The x-axis represents the name of test, whereas the y-axis represents the proportion of all strains.

The morphological, physiological and biochemical characteristics of the identified bacteria were observed and described, and the results are shown in Figure 5. Statistical data showed that among 98 species of bacteria, 81 were bacilli and 17 were cocci. Among the 838 strains, Gram-positive and Gram-negative bacteria accounted for 53% and 47%. A total of 32% of bacteria were motile; 8% of bacterial oxidase reactions were positive; 80% of bacterial contact enzyme reactions were positive; 68% of bacterial methyl red reactions were positive; 30% of bacterial V-P reactions were positive; 23% of bacterial indole reactions were positive; 26% of bacterial gelatin liquefaction reactions were positive; 41% of the bacteria reacted positively to citrate; 67% of the bacteria reacted positively to nitrate; 15% of the bacteria reacted positively to thiosulfate.

In the test of breakdown reactions for three common nutrients, nearly half of the bacteria (50%) tested positive for cellulose. In addition, only 9% of bacterial starch hydrolysis was positive and 12% of bacterial lipase reactions were positive. Only one species of bacteria can produce amylase, cellulolytic enzyme and esterase at the same time, 12 species of bacteria can produce amylase, 6 species can produce fiber-bundle-decomposing enzyme and esterase, and 43 species cannot produce these three enzymes.

#### 3.3.2. pH Gradient Culture Test

The results of the pH gradient culture test are shown in Supplementary Table S3. All bacteria were unable to grow under pH 2 and pH 11. Comparing the growth of bacteria at pH 4 and pH 9, 15 species of bacteria had similar growth conditions under the two conditions, 27 species of bacteria grew better under alkaline conditions, 6 species grew better under acidic conditions, 35 types of bacteria did not grow under acidic conditions, and three types of bacteria did not grow under alkaline conditions. The gut bacterial of *G. gratiosa* appears to be more suitable for survival in an alkaline environment than in an acidic environment.

#### 3.3.3. Drug Susceptibility Testing

**Figure 6:**
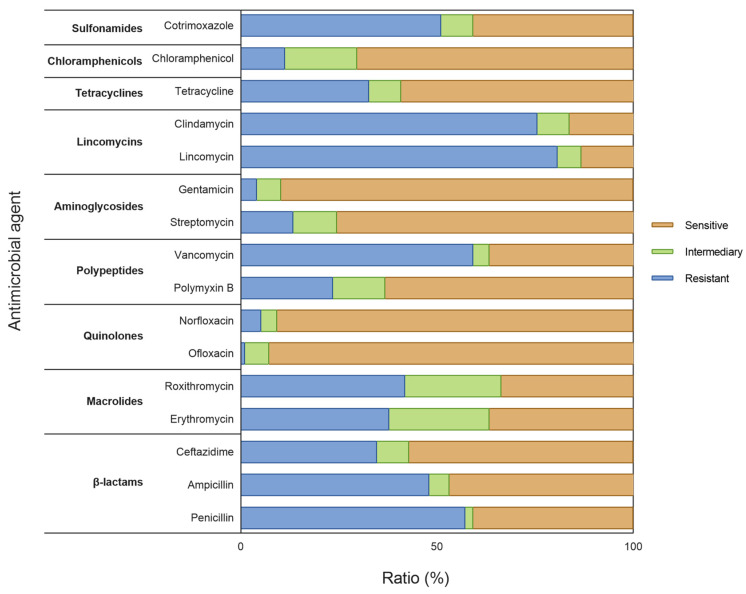
The specific results of drug susceptibility testing of 98 species of bacteria.

The specific results of drug susceptibility testing of 98 species of bacteria are shown in Figure 6 and Supplementary Table S4. Most of the gut bacteria were sensitive to quinolones and aminoglycosides, were resistant to lincomycin, and had certain sensitivity to tetracyclines, chloramphenicol, β-lactams, and macrocyclines.

The sensitivity of 98 kinds of bacteria to 16 kinds of antibiotics were investigated. There were two strains not resistant to the tested antibiotics, namely *C. glyciniphilum* and *N. dassonvillei* subsp. Dassonvillei; both species were Actinobacteria. There were seven species sensitive to 13–14 antibiotics: *P. fasciculus* and *W. chitiniclastica* (Proteobacteria), *O. kimchi*, *L. lactis* subsp. hordniae, *B. frigoritolerans*, *P. Validus* and *L. curvatus* (Firmicutes).

## 4. Discussion

Insect gut bacteria are more diverse than other parts of their bodies. Over the past decade, there have been many studies on culturable bacteria in insect guts. Research has found that insect gut bacterial flora can be dynamic and change with factors such as time and developmental stage [24]. The composition and diversity of insect intestinal flora are affected by various factors. The bacterial flora may be different in the same host species, or the same in different host species [25]. The structure of insect intestinal bacterial flora also has spatial variability, which affects the prediction of bacterial types and functions. For example, culturable bacteria in the intestines of *Anabrus simplex* will be exchanged between the host and the environment; Lactobacilliaceae were commonly found in the foregut and midgut of the host, and Enterobacteriaceae were commonly found in the hindgut [17]. The structure of insect intestinal flora is also closely related to feeding habits. The diversity of intestinal bacteria in omnivorous insects is greater than that in herbivorous and carnivorous insects. Their bodies contain a large number of intestinal bacteria, and some bacteria can form a complete functional system with the host, which can help overcome host nutritional limitations and chemical defenses [9]. The above research results showed that the bacterial flora structure in the intestine is greatly affected by regional morphological and physiological differences in the intestine. Insect intestines with relatively simple structures have lower bacterial diversity, while insect intestines with more complex structures have higher bacterial diversity.

The intestinal structure of Orthoptera insects is more complex than that of Lepidoptera and other insects, but simpler than that of Hemiptera, Hymenoptera, Coleoptera, Diptera and other insects [26]. The intestine of *G. gratiosa* is a complex micro-ecosystem with strict restrictions on the survival of bacteria. Only a small number of bacteria can adapt to the intestinal environment, resulting in a much lower diversity of intestinal microorganisms than the surrounding environment [8]. In this study, the gut of *G. gratiosa* was used as the research material. Twelve kinds of culture media were used to culture its gut homogenate in vitro. A total of 98 species of bacteria in 3 phyla, 5 classes, 11 orders, 20 families, and 45 genera were isolated. It can be seen from the culture results that the intestinal bacteria of *G. gratiosa* were rich in bacterial species, and were dominated by Proteobacteria and Firmicutes, which was consistent with the current research results on intestinal bacteria of most Orthoptera insects [19,27]. Referring to previous studies on human intestinal bacteria, it can be found that Firmicutes bacteria account for 51% of fecal microorganisms in hosts whose diet is low in fat and animal protein and rich in starch, fiber, plant polysaccharides, and is predominantly vegetarian. In contrast, for people whose diet is mainly based on animal protein, sugar, starch and fat, Firmicutes bacteria account for only 12% of fecal microorganisms, while Bacteroidetes account for 73% [28]. The phylum Proteobacteria has the largest phylogenetic composition, comprising 116 validated bacterial families, and has greatly variable morphology and versatile physiology. It is widely found in soil (36.5%) (Lauber et al., 2009), plants (62.0%) [29], seawater (57.9%) [30], and freshwater (61.3%) [31] and the atmosphere (77.9%) [32]. Obviously, these data can be used as a reference to explain the composition of the intestinal microbiota of *G. gratiosa*.

In previous research, there were a total of 25 phyla, 42 classes, 60 orders, 116 families, 241 genera and some unclassified of taxa identified from the feces of *G. gratiosa* of different developmental stages and genders raised in the laboratory; these studies used Illumina sequencing of the 16S rDNA V3-V4 region, among which Proteobacteria and Firmicutes dominate [8]. However, the results of the referenced study showed that *Kluyveria*, *Obesumbacterium*, *Buttiauxella*, *Lactobacillus* and *Hafnia* were the dominant bacterial genera in the intestine, which was different from the results of our study at the genus level. In this study, the five genera with the highest abundance of 838 culturable bacterial strains were *Klebsiella*, *Lactococcus*, *Enterococcus*, *Kluyvera* and *Pseudocitrobacter*, and among the 98 intestinal bacterial species identified, the dominant genera were *Klebsiella*, *Enterococcus*, *Bacillus*, *Acinetobacter* and *Lactobacillus*. These differences may be caused by many factors, such as bacteria that are difficult to isolate by conventional culture methods, differences between experimental samples, different compositions of feces and intestinal flora, etc. In fact, the complementarity between culture-dependent and culture-independent studies has been demonstrated, because only 15% of species detected were detected concomitantly by the two techniques [33]. We can provide a more comprehensive understanding of the composition of a microbial community by combining these two methods.

This experiment also studied the physiological and biochemical characteristics of the intestinal bacteria of *G. gratiosa*. In general, the microorganisms in the intestine of *G. gratiosa* showed physiological and biochemical characteristics consistent with its living environment. Most bacteria grew better in a slightly alkaline environment than an acidic environment, and were more suitable to live in a low-temperature environment than a high-temperature environment. More than half of the bacteria could decompose cellulose, and most bacteria could not decompose starch and fat (86.7%), which is also related to the feeding characteristics of *G. gratiosa*. Cellulose is the most widely distributed and abundant polysaccharide in nature, accounting for more than 50% of the carbon content in the plant kingdom. It mainly exists in plant cell walls, and cellulase-producing bacteria in the intestines of herbivorous insects can help the host insect decompose cellulose [34]. Many studies have been aimed at cultivating intestinal bacteria with activities such as cellulase, amylase and esterase. Nine bacterial strains were isolated from the intestine of *Plagiodera versicolora* and determined the extracellular enzyme activities of the bacterial isolates such as amylase, protease, lipase, cellulose and chitinase [35]. Yadav isolated 25 bacterial strains from the intestine of *Stromatium barbatum* through traditional bacterial isolation and culture methods, which were identified as *Staphylococcus*, *Enterobacter*, *Bacillus*, *Microbacterium*, *Pseudomonas*, *Enterococcus*, *Leucobacter*, *Brachybacterium*, *Rhodococcus* and *Ochrobactrum*. Most of the bacteria showed protease activity, followed by amylase, cellulase, pectinase and lipase [36]. Relevant studies have shown that bacteria of *Klebsiella*, *Pseudomonas*, *Bacillus*, *Streptococcus*, *Proteus*, *Citrobacter*, *Sphingomonas*, *Desulfovibrio*, *Desulphovibrio*, *Escherichia*, *Aeromonas*, and *Schewanella* have the highest probability of producing cellulase [37]. Among them, *Bacillus* is the most common and can actively participate in the degradation of compounds such as cellulose and starch [38].

The classification of the 48 bacteria with cellulase activity obtained in this experiment is shown in Figure 6. *Klebsiella*, with 12 species, was the most abundant, followed by *Bacillus*, with 5 species. Only one strain possessed cellulase, amylase and esterase activities simultaneously, namely *Klebsiella pneumoniae* subsp. rhinoscleromatis. Bacteria with the ability to degrade macromolecule nutrients such as cellulose, starch and fat obtained based on in vitro culture can serve the fields of environment and agriculture and provide new ideas for the development of microbial resources.

Antibiotics are naturally occurring or synthetic substances that can be classified into various categories, such as aminoglycosides, tetracyclines, chloramphenicol, macrolides, lincomycin, quinolones and beta-lactams [39], and can serve as a tool to explore the ecology of insect gut-related communities [40]. The sensitivity of the cultured bacteria in the gut of *G. gratiosa* differed for various antibiotics. The inhibition zones of different bacteria to the same antibiotic and of the same bacteria to different antibiotics were dissimilar, which might be attributed to the diverse substances secreted by the bacteria. In this study, two actinomycetes were resistant to all antibiotics, and several strains were resistant to only two or three antibiotics, all of which have considerable potential for further development.

Antibiotics can usually be classified according to their inhibition mechanism, including the inhibition of cell wall synthesis, cytoplasmic membrane, protein synthesis, nucleic acid synthesis, and the induction of alternative metabolic pathways [41,42]. Gram-positive bacteria are sensitive to macrolide, cephalosporins, β-lactamase inhibitor, penicillin and clindamycin drug sensitive, while Gram-negative bacteria are sensitive to aminoglycoside, polymyxin B and quinolones [43]. Most of the bacteria isolated in this study were sensitive to quinolones and aminoglycosides, somewhat sensitive to tetracycline, β-lactam, macrolides, and resistant to clindamycin. Considering that the proportion of Gram-positive bacteria and Gram-negative bacteria is approximately 1:1, the cultured bacteria in the gut of *G. gratiosa* may be more sensitive to the antibiotics that are more effective against Gram-negative bacteria.

Among the strains obtained in this study, approximately 11% (91 strains) of the bacterial 16S rRNA gene sequencing sequences had an alignment similarity lower than 98.65%, which was speculated to be a potential new species. Of these, 85 strains could be assigned to the genus level, while 6 strains could only be assigned to the family level. According to the culture results, Proteobacteria was the most isolated phylum. However, there were 42 uncharacterized strains assigned as potential new species of Proteobacteria. Therefore, the culture and isolation of gut bacteria is still a challenge that requires more in-depth exploration.

## 5. Conclusions

This study employed 12 different media to isolate and identify the culturable gut bacteria in *G. gratiosa*, determining the species of culturable microorganisms in the intestines of the grasshopper. A total of 98 species of bacteria were identified, belonging to 3 phyla, 5 classes, 11 orders, 20 families, and 45 genera. Moreover, we further investigated the physiological and biochemical characteristics and the antibiotic susceptibility of the isolates to 16 common antibiotics. These data can lay a theoretical foundation for the further exploration of the physiological function of gut bacteria of katydids and the relationship between gut bacteria and hosts, and offer support for the full utilization of gut bacteria to regulate the nutrition, growth, metabolism and immunity of the host insect.

## Figures and Tables

**Table 1:** Culture conditions used for culturing bacteria.

No.	Medium	Abbreviate
1	LB Agar solid medium	LB
2	Tryptic Soy Agar	TSA
3	MRS medium	MRS
4	Gauze’s Medium No.1, Modified	GAO
5	Nutrient Agar Medium	NAM
6	Columbia Blood Agar	BLA
7	Aeromonas Medium	AER
8	Enterococcus Agar	ENT
9	Enterobacteria Enrichment Broth	EE
10	Salmonella-Shigella Agar	SS
11	Carrot Agar	CAR
12	YCFA Medium	YCFA

## Data Availability

The data generated during this study were deposited in NCBI (Nucleotide accessions OQ405730-OQ406091).

## Supplementary Materials

The following supporting information can be downloaded at: https://www.mdpi.com/article/10.3390/insects16020123/s1, Table S1: Criteria for determining drug sensitivity of 16 antibiotics.xlsx; Table S2: The pure-culture bacteria in gut bacteria.xlsx; Table S3: The results of the pH gradient culture test.xlsx; Table S4: The specific results of drug susceptibility testing.xlsx.

## References

[1] Kashima T., Nakamura T., Tojo S. (2006). Uric acid recycling in the shield bug, *Parastrachia japonensis* (Hemiptera: Parastrachiidae), during diapause. J. Insect Physiol..

[2] Pernice M., Simpson S.J., Ponton F. (2014). Towards an integrated understanding of gut microbiota using insects as model systems. J. Insect Physiol..

[3] Erkosar B., Storelli G., Defaye A., Leulier F. (2013). Host-intestinal microbiota mutualism: “learning on the fly”. Cell Host Microbe.

[4] Allegrucci G., Sbordoni V. (2019). Insights into the molecular phylogeny of Rhaphidophoridae, an ancient, worldwide lineage of Orthoptera. Mol. Phylogenetics Evol..

[5] Zhou Z., Kou X., Qian L., Liu J. (2016). Transcriptome profile of Chinese bush cricket, *Gampsocleis gratiosa*: A resource for microsatellite marker development. Entomol. Res..

[6] Zhou Z., Shi F., Huang Y. (2008). The complete mitogenome of the Chinese bush cricket, *Gampsocleis gratiosa* (Orthoptera: Tettigonioidea). J. Genet. Genom..

[7] Su C., Chen J., Shi F., Guo M., Chang Y. (2017). Formation of the acrosome complex in the bush cricket *Gampsocleis gratiosa* (Orthoptera: Tettigoniidae). Arthropod Struct. Dev..

[8] Zhou Z., Huang H., Che X. (2022). Bacterial communities in the feces of laboratory reared *Gampsocleis gratiosa* (Orthoptera: Tettigoniidae) across different developmental stages and sexes. Insects.

[9] Xu L., Sun L., Zhang S., Wang S., Lu M. (2019). High-resolution profiling of gut bacterial communities in an invasive beetle using PacBio SMRT sequencing system. Insects.

[10] Meng Y., Li S., Zhang C., Zheng H. (2022). Strain-level profiling with picodroplet microfluidic cultivation reveals host-specific adaption of honeybee gut symbionts. Microbiome.

[11] Xu J., Sun L., Xing X., Sun Z., Gu H., Lu X., Li Z., Ren Q. (2020). Culturing bacteria from fermentation pit muds of Baijiu with culturomics and amplicon-based metagenomic approaches. Front. Microbiol..

[12] Lagier J.C., Armougom F., Million M., Hugon P., Pagnier I., Robert C. (2012). Microbial culturomics: Paradigm shift in the human gut microbiome study. Clin. Microbiol. Infect..

[13] Lagier J.C., Khelaifia S., Alou M.T., Ndongo S., Dione N., Hugon P. (2016). Culture of previously uncultured members of the human gut microbiota by culturomics. Nat. Microbiol..

[14] Prabhakar C.S., Sood P., Kanwar S.S., Sharma P.N., Kumar A., Mehta P.K. (2013). Isolation and characterization of gut bacteria of fruit fly, *Bactrocera tau* (Walker). Phytopharmaceutica.

[15] Campillo T., Luna E., Portier P., Fischer-Le Saux M., Lapitan N., Tisserat N.A., Leach J.E. (2015). *Erwinia iniecta* sp. nov., isolated from Russian wheat aphid (*Diuraphis noxia*). Int. J. Syst. Evol. Microbiol..

[16] Zhang S., Huang J., Wang Q., You M., Xia X. (2022). Changes in the host gut microbiota during parasitization by parasitic wasp *Cotesia vestalis*. Insects.

[17] Smith C.C., Srygley R.B., Healy F., Swaminath K., Mueller U.G. (2017). Spatial structure of the Mormon cricket gut microbiome and its predicted contribution to nutrition and immune function. Front. Microbiol..

[18] Zheng X., Zhu Q., Zhou Z., Wu F., Chen L., Cao Q., Shi F. (2021). Gut bacterial communities across 12 Ensifera (Orthoptera) at different feeding habits and its prediction for the insect with contrasting feeding habits. PLoS ONE.

[19] Zheng X., Zhu Q., Qin M., Zhou Z., Liu C., Wang L., Shi F. (2022). The role of feeding characteristics in shaping gut microbiota composition and function of Ensifera (Orthoptera). Insects.

[20] Lagier J.C., Dubourg G., Million M., Cadoret F., Bilen M., Fenollar F., Levasseur A., Rolain J.M., Fournier P.E., Raoult D. (2018). Culturing the human microbiota and culturomics. Nat. Rev. Microbiol..

[21] Oberhardt M.A., Zarecki R., Gronow S., Lang E., Klenk H., Gophna U., Ruppin E. (2015). Harnessing the landscape of microbial culture media to predict new organism-media pairings. Nat. Commun..

[22] Weisburg W.G., Barns S.M., Pelletier D.A., Lane D.J. (1991). 16S ribosomal DNA amplification for phylogenetic study. J. Bacteriol..

[23] Dong X., Zhou Y., Zhu H. (2023). Manual for Systematic Classification and Identification of Common Bacteria and Archaea.

[24] Augustinos A.A., Tsiamis G., Caceres C., Abd-Alla A.M.M., Bourtzis K. (2019). Taxonomy, diet, and developmental stage contribute to the structuring of gut-associated bacterial communities in tephritid pest species. Front. Microbiol..

[25] Osei-Poku J., Mbogo C.M., Palmer W.J., Jiggins F.M. (2012). Deep sequencing reveals extensive variation in the gut microbiota of wild mosquitoes from Kenya. Mol. Ecol..

[26] Engel P., Moran N.A. (2013). The gut microbiota of insects—Diversity in structure and function. FEMS Microbiol. Rev..

[27] Muratore M., Prather C., Sun Y. (2020). The gut bacterial communities across six grasshopper species from a coastal tallgrass prairie. PLoS ONE.

[28] De Filippo C., Cavalieri D., Di Paola M., Ramazzotti M., Poullet J.B., Massart S., Collini S., Pieraccini G., Lionetti P. (2010). Impact of diet in shaping gut microbiota revealed by a comparative study in children from Europe and rural Africa. Proc. Natl. Acad. Sci. USA.

[29] Redford A.J., Fierer N. (2009). Bacterial succession on the leaf surface: A novel system for studying successional dynamics. Microbiol. Ecol..

[30] Teeling H., Fuchs B.M., Becher D., Klockow C., Gardebrecht A., Bennke C.M., Kassabgy M., Huang S., Mann A.J., Waldmann J. (2012). Substrate-controlled succession of marine bacterioplankton populations induced by a phytoplankton bloom. Science.

[31] Pascault N., Roux S., Artigas J., Pesce S., Leloup J., Tadonleke R.D., Debroas D., Bouchez A., Humbert J.F. (2014). A high-throughput sequencing ecotoxicology study of freshwater bacterial communities and their responses to tebuconazole. FEMS Microbiol. Ecol..

[32] Whon T.W., Kim M.S., Roh S.W., Shin N.R., Lee H.W., Bae J.W. (2012). Metagenomic characterization of airborne viral DNA diversity in the near-surface atmosphere. J. Virol..

[33] Lagier J.C., Hugon P., Khelaifia S., Fournier P.E., La Scola B., Raoult D. (2015). The rebirth of culture in microbiology through the example of culturomics to study human gut microbiota. Clin. Microbiol. Rev..

[34] Watanabe H., Tokuda G. (2010). Cellulolytic systems in insects. Annu. Rev. Entomol..

[35] Demirci M., Sevim E., Demir I., Sevim A. (2013). Culturable bacterial microbiota of *Plagiodera versicolora* (L.) (Coleoptera: Chrysomelidae) and virulence of the isolated strains. Folia Microbiol..

[36] Yadav D.S., Ranade Y., Sawant I., Ghule S., Mhaske S. (2022). Isolation, identification and functional characterisation of bacteria associated with gut of wood feeding *Stromatium barbatum* (Fabr.) (Coleoptera: Cerambycidae) larvae. Int. J. Trop. Insect Sci..

[37] Mustafa G., Zahid M.T., Ali S., Abbas S.Z., Rafatullah M. (2021). Biodegradation and discoloration of disperse blue-284 textile dye by Klebsiella pneumoniae GM-04 bacterial isolate. J. King Saud Univ. Sci..

[38] Nelson K., Muge E., Wamalwa B. (2021). Cellulolytic Bacillus species isolated from the gut of the desert locust Schistocerca gregaria. Sci. Afr..

[39] Acar J.F. (1977). Classification of antibiotics. Rev. Prat..

[40] Robinson C.J., Schloss P., Ramos Y., Raffa K., Handelsman J. (2010). Robustness of the bacterial community in the cabbage white butterfly larval midgut. Microb. Ecol..

[41] Culp E.J., Waglechner N., Wang W., Fiebig-Comyn A.A., Hsu Y.P., Koteva K., Sychantha D., Coombes B.K., Van Nieuwenhze M.S., Brun Y.V. (2020). Evolution-guided discovery of antibiot-ics that inhibit peptidoglycan remodelling. Nature.

[42] Kapoor G., Saigal S., Elongavan A. (2017). Action and resistance mechanisms of antibiotics: A guide for clinicians. J. Anaesthesiol. Clin. Pharmacol..

[43] Kim J., Ahn J. (2022). Emergence and spread of antibiotic-resistant foodborne pathogens from farm to table. Food Sci. Biotechnol..

